# An Antimicrobial Treatment Assessment of *Serratia marcescens* Bacteremia and Endocarditis

**DOI:** 10.3389/frabi.2022.942721

**Published:** 2022-06-29

**Authors:** Douglas Slain, Catessa Howard, C. Garret Cooper

**Affiliations:** ^1^Department of Clinical Pharmacy, School of Pharmacy, Section of Infectious Diseases, School of Medicine, West Virginia University, Morgantown, WV, United States; ^2^Department of Pharmacy, West Virginia University (WVU) Medicine, Morgantown, WV, United States; ^3^Department of Medicine, West Virginia University School of Medicine, Morgantown, WV, United States

**Keywords:** antibacterials, *Serratia marcescens*, AmpC β-lactamase, bacteremia, endocarditis

## Abstract

We assessed the treatment of *Serratia marcescens* bacteremia and endocarditis in one of the largest single center studies. We could not identify an advantage with any particular antibiotic treatment regimen in this study. Induction of AmpC or selection of ESBL organisms was not displayed by any of the organisms.

## Introduction

*Serratia marcescens* is an opportunist gram-negative bacilli that has been an occasional cause of healthcare-associated infection and as a cause of bacteremia and endocarditis in people who inject illicit drugs (PWID) (Phadke and Jacob, 2016). This bacteria remains somewhat of a mystery, and decisions about the treatment of *S. marcescens* infections are difficult given the limited clinical study data available and concerns about the potential for inducible multidrug resistance. *Serratia marcescens* has the ability to produce inducible AmpC β-lactamase and may even acquire extended-spectrum β-lactamase (ESBL) (Mahlen, 2011). Compared to other more virulent bacteria, *S. marcescens is* unlikely to overexpress AmpC β-lactamase (Tamma et al., 2021). Therefore, using broad-spectrum antibiotics that are typically stable to AmpC producing bacteria (carbapenems or cefepime) may not be required to treat *S. marcescens* infections successfully (Harris et al., 2016; Tamma et al., 2021). In terms of antimicrobial stewardship, use of carbapenem-sparing antibiotic regimens has become an attractive option as rates of carbapenem-resistant Enterobacteriacea (CRE) have increased worldwide (Tan et al., 2020).

Our university hospital has experienced a higher exposure to patients with *S. marcescens* bacteremia compared to many similar institutions in the United States. The higher rate may be fueled in particular by intravenous drug use practices and possibly environmental factors in the Appalachian region of the United States. It has been postulated that this particular organism occurs in the PWID population due to (A) recurrent exposure to medications directed at gram positive organisms, (B) exposure to hospitals, or (C) the practice of diluting the narcotic with water (Rosenblatt et al., 1973). Our clinicians have had to rely on their own clinical judgments for treating *S. marcescens* bacteremia and endocarditis, given the lack of good evidence-based guidelines. This brief study was designed to assess *S. marcescens* bacteremia/endocarditis treatment selection and outcomes in one of the largest collections of *S. marcescens* bacteremia/endocarditis from a single academic medical center.

## Methods

This observational study included adult patients admitted to the University Hospital between 2017 and 2019 with *S. marcescens* bloodstream and/or endocarditis infections. Patients were excluded from the analysis, if they had a concomitant infection with another gram-negative organism, or had a previous *S. marcescens* infection within the previous 6 months. Our evaluation was designed to identify clinician-selected antibiotic therapy, compare clinical outcomes associated with different antibiotic regimens, evaluate how care differed in PWID patients vs. others, and identify factors associated with obtaining infectious diseases expert consultations. Phenotypic susceptibility patterns were also compared.

In addition to the initial positive bloodstream isolates, all subsequent isolates were assessed for clearance and/or recurrence. Clearance was defined as negative cultures after the start of antibiotic therapy (with no subsequent positive cultures during hospitalization). Recurrence was defined as a positive culture occurring after treatment initiation, negative cultures and hospital discharge. Susceptibility testing was performed primarily using standardized Vitek 2 automated susceptibility testing (bioMérieux, Inc. Durham, NC) with infrequent use of Kirby-Bauer disc diffusion testing for some agents. Patients' electronic medical records were followed for 90 days from the time of the first *S. marcescens* culture. Biostatistical analysis which included multi-logistic regression was performed using JMP Software, Version 14 (SAS Institute, Cary NC). This study was granted exempt status by the WVU Institutional Review Board due to its observational design.

## Results

Two hundred-twenty unique patients had positive blood cultures for *S. marcescens* during the study assessment period. Of these, 43 patients met study inclusion/exclusion criteria and their characteristics are summarized in Table 1. Endocarditis was diagnosed in 13 (30.2%) of patients. Six patients had prosthetic heart valves in place prior to developing *S. marcescens* infections. Twenty-four (55.8%) of patients admitted to using intravenous drugs of abuse in their recent history, and 28 patients (65.1%) received infectious diseases specialist consults while hospitalized. When all characteristics were compared by multi-logistic regression, only PWID (*p* = 0.004), endocarditis (*p* = 0.0002), sepsis (*p* = 0.022), elevated hepatic enzymes (*p* = 0.030), and surgical intervention (*p* = 0.003), were independently associated with obtaining an Infectious Diseases Expert Consult.

**Table 1:** Patient characteristics (*n* = 43).

**Characteristic**	**Number (%) unless specified**
Age (years) mean ± standard deviation	48.7 ± 15.0
Gender (female/male)	19/24
BMI (kg/m^2^) ± standard deviation	29.4 ± 8.95
Intravenous drug abuse	24 (55.8)
Endocarditis	13 (30.23)
Prosthetic heart valve	6 (13.95)
β-lactam allergy reported	9 (20.93)
Serum creatinine (mg/dL) mean (range)	0.92 (0.4–6.78)
Abnormal hepatic enzymes	7 (16.28)
Septic at diagnosis	17 (39.53)
Gram-positive cocci or Candida isolated in blood	10 (23.26)
Had infectious diseases consult	28 (65.12)
Valve surgery as part of treatment	9 (20.93)

**Figure 1:**
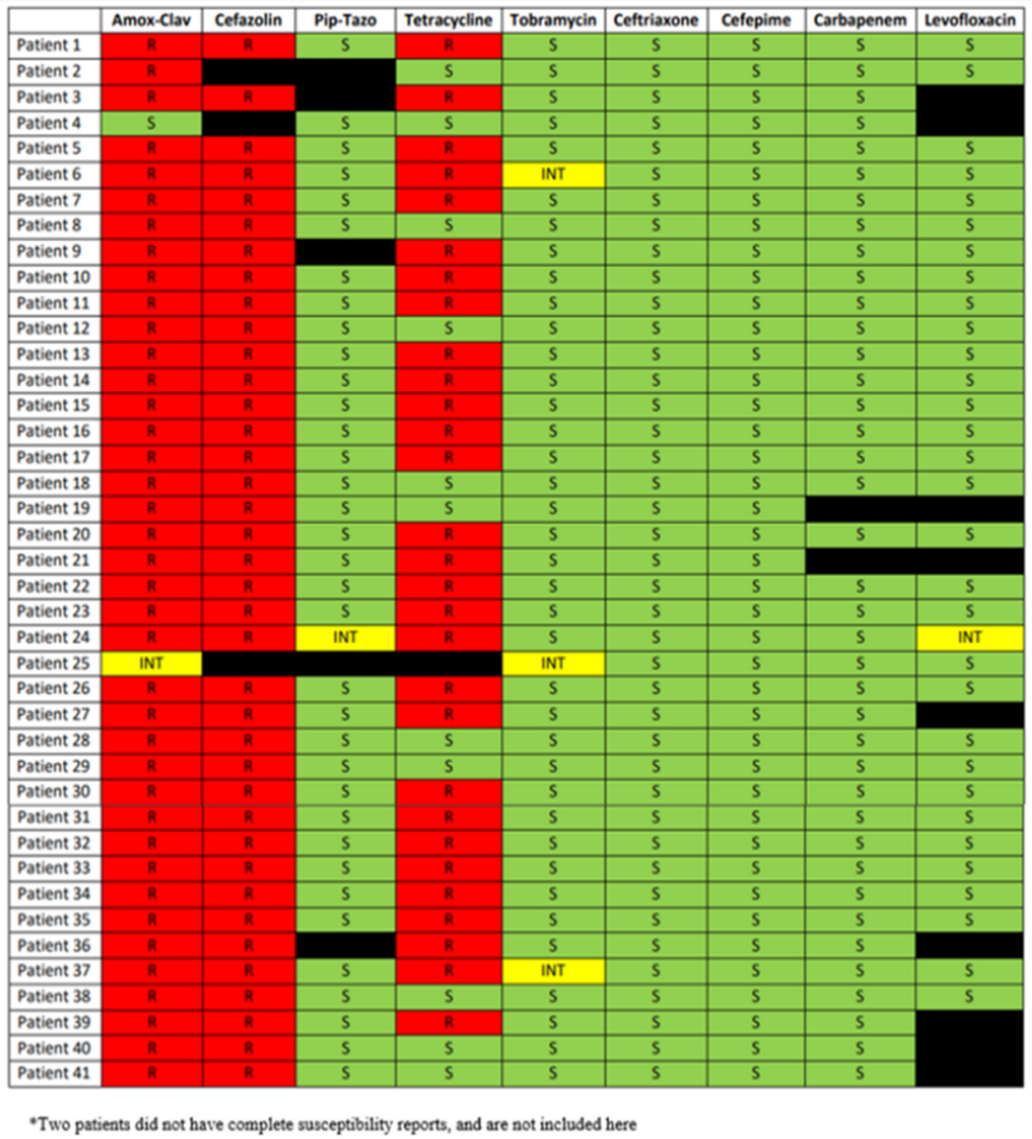
Phenotypic antimicrobial susceptibility for 41 patients.

Full phenotypic antimicrobial susceptibility reports were available for 41 patients (see Figure 1). Automated phenotypic susceptibility testing did not identify either ESBL production or AmpC expression in any of the isolates. The few repeat positive cultures seen in this study did not display a difference in susceptibility from the baseline susceptibility report. The susceptibility patterns for the *S. marcescens* isolates were very consistent and displayed resistance to amoxicillin-clavulanate and cefazolin, while displaying susceptibility to tobramycin, ceftriaxone, cefepime, carbapenems, and levofloxacin. Tetracycline resistance was seen in 72.5% of the isolates.

Table 2 highlights the treatment selection and clinical responses. The most common treatment regimen was cefepime (44.2%) followed by meropenem (13.9%) or cefepime + aminoglycoside or fluoroquinolone (13.9%). Piperacillin-tazobactam was used in 11.6% of patients. Some patients had “mixed” regimens that included limited days of particular antibiotic(s) with switches or additions to other therapies over the course of their treatment (11.6%).

**Table 2:** Selected treatments and clinical responses (*n* =43).

**Gram-negative treatment**	**Number** **(%)**	**Clinical response**		
		**Resolved/cured**	**Recurrence**	**Died of underlying condition**
Cefepime	19 (44.2%)	13	3	3
Carbapenem (Meropenem)	6 (13.9%)	3	0	3
Piperacillin-tazobactam	5 (11.6%)	3	2	0
Cefepime + aminoglycoside or fluoroquinolone	6 (13.9%)	5	0	1
Meropenem + aminoglycoside or fluoroquinolone	2 (4.6%)	1	1	0
Mixed therapies*	5 (11.6%)	5	0	0

* Mixed therapies accounted for regimens with limited days of particular antibiotic(s) due to switches or additions to other therapies over the course of their treatment.

Combination therapy was only recommended for endocarditis and with an Infectious Diseases Consult. When all characteristics were compared by multi-logistic regression, only PWID (*p* = 0.004), endocarditis (*p* = 0.0002), sepsis (*p* = 0.022), and surgical intervention (*p* = 0.003), were independently associated with obtaining an Infectious Diseases Consult. Most patients (90.7%) cleared their blood stream within 48 h of antibiotic start. Recurrence rates were generally low, with six cases seen overall. The patients with recurrences were treated with either a carbapenem or combination therapy. The patients with recurrences did receive therapies that were susceptible per microbiology lab report. Seven patients died within 60 days of their first positive *S. marcescens* culture, but the cause of death was attributed to underlying conditions in those cases.

## Discussion

Our brief study provides some unique observations that can add to the thin patchwork of available information about *S. marcescens* bacteremia and endocarditis treatment. Our data comes from one of the largest single center *S. marcescens* bacteremia/endocarditis data sets. Evidence-based guidelines were not available at the time of this study to guide clinicians on the treatment of *S. marcescens* bacteremia or endocarditis. A brand new IDSA “guidance document” has just been released to provide clinicians with some guidance for the treatment of AmpC producing organisms (Tamma et al., 2021). That guidance document provides “suggested approaches” based on clinical experience, expert opinion, and a review of the available literature, rather than using evidence-based grading criteria like GRADE or the US Public Health criteria (Kavanagh, 2009). The document was only able to provide limited guidance on *S. marcescens* bacteremia and endocarditis treatment, due to the paucity of good clinical data in the medical literature.

The majority of antibiotic courses used at our hospital for *S. marcescens* bacteremia and endocarditis provided carbapenem-sparing treatment with a low rate of recurrence. Cefepime alone or in combination made up the majority of courses. Cefepime is often a preferred agent due to its stability against AmpC β-lactamase and low potential for AmpC induction (Tamma et al., 2021). Cefepime is generally well tolerated by most patients, but there are increasing concerns about the over use of this agent and its drug-associated neurotoxicity (Payne et al., 2017).

Piperacillin-tazobactam was the main therapy in five patients, despite the concern for any inducible AmpC expression. Piperacillin-tazobactam has been used with success in bacteremic infections with AmpC producing organisms (Harris et al., 2016; Tan et al., 2020). Use of piperacillin-tazobactam against *S. marcescens* is an option in the new IDSA guidance document, but the authors recommend caution in high inoculum infections like endocarditis or other serious infections (Tamma et al., 2021). Use of piperacillin-tazobactam as a carbapenem-avoidance strategy has been an area of active research in recent years. The MERINO-2 study group was not able to find a difference between piperacillin-tazobactam and meropenem among patients with bloodstream infections with AmpC producing organisms, unfortunately, there were only five Serratia infections in that study (Stewart et al., 2021). Similarly, a recent meta-analysis of bloodstream infections with AmpC-producing organism also failed to show a difference between piperacillin-tazobactam and carbapenems among studies with modest cases of *S. marcescens* infections (Cheng et al., 2017). In our study, we did see two recurrences in the five patients treated with piperacillin-tazobactam, but this subset is too small to denigrate piperacillin-tazobactam. Nonetheless, it does provide reason for additional study.

An interesting observation from our study was that combination therapy was only used for endocarditis treatment and with an Infectious Diseases Consult. This approach was deemed “reasonable” for non-HACEK gram-negative (not specific to *S. marcescens*) endocarditis in the most recent American Heart Association (AHA) endocarditis guidelines (Baddour et al., 2015). This may suggest that infectious diseases specialists are more familiar with the AHA guidelines than non-infectious diseases specialists. The level of evidence for that reasonable recommendation in the AHA guidelines was at the expert opinion level.

One of the encouraging observations from our study is that almost all patients experienced a quick bacteremia clearance rate (<48 h). This may reflect the oft-suggested low pathogenicity of *S. marcescens*, and could promote the use of shorter courses of intravenous antibiotics for this organism (Yu, 1979; Chotiprasitsakul et al., 2018; Yahav et al., 2019).

## Conclusions

We could not identify an advantage of any particular antibiotic treatment regimen in this study. This may be due to the small sample size of infections with this relatively infrequent pathogen. Nonetheless, induction of AmpC β-lactamase or selection of ESBL organisms was not displayed by any of the organisms. Prospective randomized studies should be performed to better evaluate antimicrobial treatment options and duration for *S. marcescens* bacteremia and endocarditis.

## Data Availability Statement

The raw data supporting the conclusions of this article will be made available by the authors, without undue reservation.

## Ethics Statement

The study involving human participants was reviewed and approved by West Virginia University Institutional Review Board. Written informed consent for participation was not required for this study in accordance with the national legislation and the institutional requirements.

## Author Contributions

DS, CH, and CC contributed to the conception, study design, data analysis, and manuscript preparation and revision. DS and CH performed the data collection. DS worked with biostatistician to perform statistical analysis. All authors contributed to the article and approved the submitted version.

## Conflict of Interest

The authors declare that the research was conducted in the absence of any commercial or financial relationships that could be construed as a potential conflict of interest.

## Publisher's Note

All claims expressed in this article are solely those of the authors and do not necessarily represent those of their affiliated organizations, or those of the publisher, the editors and the reviewers. Any product that may be evaluated in this article, or claim that may be made by its manufacturer, is not guaranteed or endorsed by the publisher.
